# Functional Changes and Driving Performance in Older Drivers: Assessment and Interventions

**DOI:** 10.3390/geriatrics1020012

**Published:** 2016-05-20

**Authors:** Melanie Karthaus, Michael Falkenstein

**Affiliations:** 1 Leibniz Research Centre for Working Environment and Human Factors at TU Dortmund (IfADo), Dortmund D-44139, Germany; 2 Institute for Working, Learning, and Aging, Bochum D-44805, Germany

**Keywords:** aging, mobility, older drivers, cognitive functions, driving assessment, screening, cognitive training, on-road driving, driving simulator

## Abstract

With the increasing aging of the population, the number of older drivers is rising. Driving is a significant factor for quality of life and independence concerning social and working life. On the other hand, driving is a complex task involving visual, motor, and cognitive skills that experience age-related changes even in healthy aging. In this review we summarize different age-related functional changes with relevance for driving concerning sensory, motor, and cognitive functions. Since these functions have great interindividual variability, it is necessary to apply methods that help to identify older drivers with impaired driving abilities in order to take appropriate measures. We discuss three different methods to assess driving ability, namely the assessment of (i) functions relevant for driving; (ii) driving behavior in real traffic; and (iii) behavior in a driving simulator. We present different measures to improve mobility in older drivers, including information campaigns, design of traffic and car environment, instructions, functional training, and driving training in real traffic and in a driving simulator. Finally, we give some recommendations for assessing and improving the driving abilities of older drivers with multi-modal approaches being most promising for enhancing individual and public safety.

## 1. Older Drivers and Traffic Security

In all countries the percentage of older people is strongly increasing. By 2050 the number of people aged 80 and older will most probably triple in the OECD countries and a third of the population will be older than 65 years [1]. This development also leads to a rising number of older people who want or have to stay mobile.

In this context driving one’s own car is of great importance because it allows older people a self-determined mobility. Particularly in rural regions, in which an above-average proportion of the elderly are living, driving is of utmost importance since local public transport is scarce. However, even in congested urban areas with good public transport many older people prefer driving their own car due to fear of assaults in buses or trains [2].

Mobility is an essential attribute of quality of life in older people [3]. It can be broadly defined as the ability to move within community environments that expand from one’s home, to the neighborhood and to regions beyond [4]. In the holistic concept of Webber *et al.* [4] mobility is portrayed through five fundamental categories of determinants (cognitive, psychosocial, physical, environmental, and financial), with gender, culture, and biography (personal life history) conceptualized as critical cross-cutting influences. The restriction of mobility can lead to a decrease in social activities and in turn to an impairment of cognitive status [5] and mental health, such as higher depressiveness [6]. A study with more than 1500 subjects showed that people who stopped driving a car for more than six months had a five-fold higher risk for permanent care than active drivers, independent of confounding factors such as health state [7]. In a study of Edwards *et al.* [8], non-drivers were much more likely to die than drivers during the subsequent years. Public transport is not a good alternative because of restricted opportunities in rural areas. As pointed out by Mollenkopf *et al.* [9], older people perceived increased losses in the array of mobility experiences and decreasing satisfaction with mobility opportunities. Hence stopping driving one’s own car impairs mobility, as well as cognition and health, in the elderly and should be avoided whenever possible.

In addition, renouncing driving does not mean higher traffic security for the elderly. Hakamies-Blomqvist *et al.* [10] compared the number of fatal accidents in Finland, which requires drivers aged 70 and above to undergo medical tests to renew their driving license, and in Sweden, a country without such tests. No advantage was found in the Finnish system concerning the reduction of accident rates. In fact, the percentage of fatal accidents with pedestrians aged 70 and above was even higher in Finland than in Sweden. The authors argued that a driving restriction due to test failure can lead older people to use less safe modes of mobility, e.g., as pedestrians, which makes them more vulnerable. Similarly, in a more recent study, Siren and Meng [11] compared the rate of fatal accidents in Denmark before and after the introduction of a medical plus a short cognitive screening test for elderly drivers. Again no reduction of accidents was found in the period after test introduction; instead an increase of fatal accidents in older pedestrians and cyclists was observed. A study of Redelmeier *et al.* [12] showed that physicians’ warnings to patients who are potentially unfit to drive revealed a small reduction of hospital admissions for road accidents but also substantially increased the number of admissions for depression. In addition, stopping driving leads to a decrease of outdoor activities [13] and hence of social interactions and cognitive challenges, which keep the elderly cognitively fit. These results clearly show that restricting driving for the elderly is not a good solution in terms of either traffic security or the health and wellbeing of older people. Rather, measures should be taken that find a tradeoff between security and mobility to preserve driving in that group.

Certainly, the medical or cognitive screenings administered in Sweden and Denmark are obviously not well suited to tackle the physical, sensory, and cognitive skills that are crucial for driving in older people. The cognitive tests used in Denmark are a short version of the Mini-Mental State Examination (MMSE) [14] and the clock drawing test, which are used for dementia diagnosis. Such tests are not sensitive and hence not suited for assessing cognition in healthy older people. Moreover, they do not test those cognitive skills that are relevant for driving, such as attention and multitasking. Hence, more suitable cognitive tests that address those skills relevant to critical driving situations for older drivers are necessary. Such tests should contain most of these skills and should be adapted to the driving situation. The issue is, however, to what degree such tests can predict driving behavior. We will come back to this issue later.

However, good driving depends not only on skills but also on factors like self-rating, overconfidence, risk acceptance, sensation-seeking, *etc.* [15]. In a study of Ross *et al.* [16], most older drivers (85.14%) rated themselves as either good or excellent drivers regardless of their actual previous citation or crash rates. Drivers who overrate their competencies are more dangerous than those who acknowledge their difficulties [15]. Such discrepancies can be observed during cognitive testing [17] or real driving. Hence, leaving the decision to continue driving to the driver himself appears insufficient. Rather, a combination of self-rating assessments, optimized functional tests, and the monitoring of drivers at their task should be better suited to estimate the driving competence of older drivers.

The participation of seniors in accidents is altogether lower (about 12%) than their share in the population, which is about 20% in Germany [18]. However, when kilometers driven are taken into consideration, the accident rate of older drivers is relatively high and similar to the rate of very young drivers (e.g., [19]). In particular, drivers aged 75 years and above who drive fewer than 3000 km per year exhibit the highest accident risk [20]. In Germany the majority of seniors involved in an accident are primarily responsible for the accident; this applies to about 75% of drivers aged 75 and above [18]. A more detailed analysis of the causes of accidents with seniors shows that there are typical situations that are obviously difficult for seniors and induce accidents. These are mainly giving right of way, turning, and driving backwards, particularly in difficult or unexpected situations such as complex crossroads. Such age-typical driving errors are most probably due to age-related deterioration of sensory and cognitive functions (e.g., [21]). Due to experience and compensation strategies, such deterioration usually does not lead to accidents, but may increase the accident risk for older drivers. Accident rates or the registration of driving errors by fellow passengers are only the tip of the iceberg. Much more often, near-accidents probably happen that do not show up in any statistics. Hence there is clearly some risk and it appears necessary to use more suitable methods to test the driving ability of older drivers (e.g., [22]) rather than simple screenings, as used in the study of Siren and Meng. Even though there have been suggestions in Germany that general practitioners should use better tests for screening [3,23], this has not yet been realized.

## 2. Age-Related Functional Changes with Relevance for Driving

Complex tasks like driving require different sensory, motor, and cognitive functions und their interaction. With increasing age changes in perceptive (e.g., [24]), motor (e.g., [25]), and cognitive functions (e.g., [26]) can be reliably observed in laboratory settings. Such changes may also have an impact on everyday tasks, in particular under time pressure. Driving is such a task, hence driving competence may be impaired due to age-related functional changes. Indeed, as pointed out by Anstey *et al.* [27], cognitive and vision factors explained 83%–95% of age-related variance in the capacity to drive safely.

### 2.1. Sensory Functions

Age-related impairments of vision are most relevant for driving, because 80%–90% of traffic-relevant information is taken in via the eyes [28]. Normal aging is associated with structural changes of the eye that lead to reductions in visual acuity and contrast sensitivity and increased glare sensitivity [29]. Acuity can be compensated for with glasses, while the other problems cannot, which is linked to an increased risk for the elderly during night driving. Peripheral vision is reduced with increasing age (e.g., [30]). This can lead to problems in detecting relevant traffic stimuli in the periphery (e.g., [15]). A reduced binocular field has been shown to significantly impair certain aspects of driving performance in a driving course [31]. Interestingly, older drivers with severe impairment in the lower or left region of the driving visual field are more likely to have a history of collision involvement [32]. However, it is still unclear whether this impairment is related to the visual field restriction or to reduced visual processing speed and attention shift. We will come back to this issue later.

In addition to such normal changes, certain eye diseases that impair driving security become more frequent with increasing age [33]. One such disease is eye cataracts, which affect more than 50% of the population over 65 [34]. Accident risk due to cataracts is more than twice that of healthy drivers [35]. A further common eye disease of older people is glaucoma, which is related to enhanced intraocular pressure and nerve lesions. The global prevalence of glaucoma for the population aged 40–80 years is 3.54% [36]. Glaucoma leads to impairments of the visual field and causes a 1.7- to 5.2-fold increase in accident risk [37]. In conclusion, drivers should be screened not only for acuity but for subtle visual deficits and eventually advised to avoid situations with insufficient light.

Hearing is also strongly impaired due to normal aging but assumed to be less significant for traffic security [37]. However, strong auditory impairment can lead to the missing of important traffic noises and warning signals (e.g., [38]).

### 2.2. Motor Functions

Age-related motor changes may influence driving ability and general mobility [39].With increasing age, muscle strength strongly diminishes, and speed of movement is reduced [40]. This can lead to a slowing of emergency maneuvers with the steering wheel or a prolongation of braking time [39]. Further, trunk and neck flexibility, which is essential for looking back during driving, is massively reduced with age, leading to insufficient detection of targets in the back view [41]. Overlooking important targets approaching from behind certainly increases accident risk.

Motor coordination and dexterity also deteriorate with rising age and are already worse in late middle age (e.g., [42]). The ability to stay in one’s lane during subtle influences like a side wind is already slightly impaired in late middle-aged adults [43]. Hence complex and fine-grained movements, which are sometimes necessary during driving, in particular in unexpected and emergency situations, are probably impaired in the elderly. Interestingly, the rate of falling was associated with accident involvement of older drivers [44]. However, some of the difficulties in motor function can be overcome by muscle and flexibility training on the one hand and by driving assistance systems, such as improved mirror systems and cameras or parking assistants, on the other hand [39].

### 2.3. Cognitive Functions

Certain cognitive functions that are relevant for driving also show a clear deterioration with increasing age in healthy adults; some functions such as visual search are already considerably impaired in middle-aged persons (e.g., [45]). Surprisingly, cognitive problems are largely overlooked in prevailing tests of driving ability in elderly adults. In particular, functions that reflect fluid intelligence deteriorate (e.g., [46]). Such functions are important for the solution of problems and coping with unexpected situations. In particular, the so-called executive functions, which control lower-level functions, are highly relevant for driving. These functions control attention supporting, e.g., visual search and attention switch, the inhibition of irrelevant information and inadequate responses, the management of multiple tasks, and the monitoring of one’s own performance.

Numerous studies suggest older adults have a problem inhibiting inappropriate information and actions. In a driving context, we could show that the elderly process irrelevant stimuli as intensively as relevant ones [47]. Such excessive processing of irrelevant information binds too many resources and can lead to cognitive overload. In traffic situations with a complex environment and multiple distractors, such as at busy intersections, this “filtering” problem of older people can enhance the risk of accidents [48].

A further cognitive function that can be impaired in higher age is the distribution and flexible control of visual attention. Visual attention involves distribution, search, selection, and switching, and plays an important role in the driving risk among older drivers [49]. For example, the division of attention across the entire space, which is mandatory for safe driving, is impaired in older age. In one of our laboratory studies subjects had to attend to targets with two possible locations (divided attention) *vs.* one fixed location (focused attention). While the performance of the young subjects hardly differed between the conditions, late middle-aged subjects showed increased reaction times and error rates under divided rather than focused attention [50]. This suggests impaired performance in the elderly in real situations where the location of a possible threat is unknown. Indeed, the elderly perform consistently worse in the useful field of view (UFOV) task, in which subjects have to perform a focal task (such as observing the traffic ahead) and a peripheral task (detecting the location of a peripheral stimulus) [51]. However, it has to be stressed that the performance in the UFOV alone is not a good predictor of driving behavior for the general population. A further driving-relevant function is visual search, *i.e.*, the quick detection of a relevant stimulus (such as a specific traffic sign) among multiple similar but irrelevant stimuli [52]. With increasing age, search time rises and error rate (*i.e.*, missed targets or false alarms) increases (e.g., [53]). Already middle-aged healthy and highly motivated subjects show clear search impairments compared to young people [45]. Hence in real traffic important traffic signs or critical targets and threats are possibly detected later or not at all by older drivers (e.g., [54]). Additionally, switching attention between different tasks, which is often the case during driving, is impaired with higher age [55].

Closely related to divided attention, the performance of dual or multiple tasks, which is also a variant of task switching, is impaired in higher age (e.g., [56]), in particular when sensorimotor and cognitive tasks have to be managed as subtasks (e.g., [43]). Driving a car is a typical example of such multitasking since it requires steering and operating the car, observing the traffic, being aware or predicting critical situations, as well as planning, executing, and adapting one’s own behavior [57]. In studies of our own lab we could show that older drivers exhibit strong deficits in very complex situations such as simultaneous tracking, attention to specific stimuli, and braking (e.g., [58]).

Despite those possible impairments of cognitive functions and related problems in certain traffic situations, poor performers in tests generally overestimate their performance [59]. Likewise, elderly drivers with poor driving performance rated their driving as good to excellent [15]. Those that perceive themselves as good drivers are not likely to be motivated to change their driving habits and behavior [60]. In addition, even when drivers at risk for crashes reduce their driving, they are still disproportionately involved high accidents [61], hence self-regulation appears to be an insufficient approach to reduce crash risk. On the other hand, there also might be some people who underestimate their performance and therefore avoid driving. Unfortunately, this may prevent further practice and automating sensorimotor responses.

It has to be emphasized that all those sensory, motor, and cognitive changes underlie a massive intra- and interindividual variability, which is influenced by a multitude of external and internal factors independent of age *per se* (e.g., [62]). In addition, despite impaired performance in laboratory tasks the elderly often behave adequately in real life, such as in traffic. This is due to the fact that in routine traffic situations mainly automated processes are necessary, which show no age-related decline. Only in unexpected and complex situations are cognitive, and in particular executive, functions needed. Consequently, it is in exactly those situations that some older drivers have problems. In addition, with increasing age many people are increasingly using compensation strategies such as selection of well-known routes or more rigorous preparation, which can buffer deficits successfully. Hence sensory and cognitive performance in tests does not predict driving competence *per se*, but can help to screen for older people with enhanced risk.

## 3. Assessment of Functions Relevant for Driving

As pointed out above, visual, motor, and specific cognitive functions are crucial for driving in unexpected and complex situations. Meaningful visual tests target visual acuity, contrast, mesopic vision, and peripheral vision. Usually only visual acuity is measured because it is very easy to test for; in Germany a visus of 0.5 is set as the minimum [63]. Contrast and mesopic vision are even more important for driving, but difficult to measure and rarely tested. For contrast vision the Pelli-Robson or MARS contrast sensitivity test can be used [64,65]. Static peripheral (in particular horizontal) vision can be measured with a perimeter, which is also rarely applied. A composite score based upon peripheral vision, visual acuity, and contrast sensitivity was found to be significantly related to increased crash rate (per mile driven) for drivers 66 years of age or older [66]. Hence not only visual acuity but the more important mesopic and peripheral measures should be assessed to ensure proper vision for driving in older age. A further important function relevant for detecting moving hazards is the perception of subtle movements. In older adults Lacherez *et al.* [67] found a decreased ability to perceive deviations in motion in driving videos, which was related to their ability to detect the moving hazards.

As mentioned above, driving-relevant motor functions are motor speed, coordination, balance, and trunk and head flexibility. Motor speed can be measured with simple reaction tasks that contain a sensory component. Both can be disentangled with electrophysiological measures (e.g., [25]), which are of course rarely applied in traffic psychology or medicine. Depending on the motor task, movements of older adults take 20 to 500 ms longer than in young adults (e.g., [25]). Not surprisingly, increased foot reaction time was significantly associated with crashes in a linear fashion [68]. Further speed and balance tests that have been applied in traffic research [22] are the grooved pegboard test [69] and the functional reach test. In the former test subjects have to insert pegs into holes oriented in different directions as fast as possible. In the latter, subjects have to lean forward as far as possible from a standing position without losing balance [70]. However, in the study of Emerson and colleagues [22], only the pegboard test was predictive of driving cessation. In general, however, the relationship between motor problems and accidents is rather weak [39] for a variety of reasons. First, the lower flexibility can be supported by information and support systems, as mentioned above. Also, experienced older drivers might use a compensation strategy to anticipate hazardous conditions rather than relying on quick reflexes to react to them. Finally, motor tests are not easy to apply in a routine test setting.

Driving-relevant cognitive functions can be measured with paper and pencil tests or better with computer-based psychometric tests. In the Emerson study, mainly cognitive test scores were predictive for crashes. A widely used paper and pencil test for general cognitive status is the Mini-Mental State Examination [14]. However, in normal samples of older adults the MMSE has strong ceiling effects. Hence, not surprisingly, performance on the MMSE was not found to be associated with either self-reported [71] or recorded [68] crashes. A further test for general cognitive status is the Montreal Cognitive Assessment (MoCA) [72]. The MoCA is much more adapted to cognitive skills necessary for driving and has better psychometric properties [73]. In a recent study Hollis *et al.* [74] showed a significant relationship between MoCA score and on-road outcome in individuals with, but not without, cognitive impairment. The authors conclude that for individuals with a pre-established diagnosis of cognitive impairment, the MoCA is a useful screening tool for driving safety.

Tests of visual attention, even simple number cancellation tests, are highly predictive of older drivers’ accidents [71,75]. One of the most widely used tests of peripheral and divided attention as well as processing speed is the abovementioned Useful Field of View (UFOV) Test [51]. Many studies showed that older people perform worse in the UFOV task (e.g., [76]), and high UFOV scores predict driving cessation [22] and accident risk (e.g., [77]). In particular, poor UFOV performance was associated with increased crash risk of about 100% in two prospective reports [78,79]. Concerning specific driving problems, the UFOV predicted blind-spot errors and errors on dual carriageways in an on-road driving test [75].

A further important test that is used in traffic research is the Trail Making Test (TMT) [80]. It has two parts: in the easier part (TMT A) the subjects have to search and tie successive numbers on a screen or sheet of paper; in the more difficult part (TMT B) they have to do the same alternatively with numbers and letters in alphabetical order. While TMT A predominantly measures visual search and working memory, TMT B measures attention shifting in addition to increased demands on working memory. Some studies found the TMT—particularly the TMT B score minus the TMT A score—predictive of crashes (e.g., [22,77]). However, more recent studies questioned the utility of the TMT. Dobbs and Shergill [81] showed that TMT A and B outcomes are most likely inaccurate in older drivers whose driving competency has declined to an unsafe level. In a large study with more than 400 older drivers, Vaucher *et al.* [82] showed that the TMT, although predictive of poor driving, is not specific enough to justify driving cessation without complementary investigations on driving behavior.

This shows that single tests of specific cognitive functions are not suited to estimate driving behavior. However, a combination of tests appears to be more fruitful. For example, McKnight and McKnight [83] administered a comprehensive battery of visual, cognitive, and psychomotor tests as well as a structured road test to 407 elderly drivers, the majority of whom had been referred to licensing agencies because of traffic incidents. A total score based upon all ability measures showed a sensitivity of 80% of incident-involved, misidentifying only 20% of the incident-free drivers, which means a specificity of 80%. In a recent study Bowers *et al.* [84] showed that the combination of the best four tests of a small visual–cognitive battery was able to identify at-risk drivers with 95% specificity and 80% sensitivity (0.91 AUC). Wood *et al.* [85] showed that a combination of three tests from the vision, cognitive, and motor domains, including motion sensitivity, color choice reaction time, postural sway on a compliant foam rubber surface, and a self-reported measure of driving exposure, was able to classify participants into safe and unsafe driver groups with a sensitivity of 91% and a specificity of 70%. However, Wood *et al.* tested the validity of their model on the same sample the model was derived from, which leads to some chance results. These results therefore need to be confirmed in a second sample. Recently Vaucher *et al.* [86] developed a cognitive test battery (MedDrive) consisting of four tests including a new variant of the UFOV, an attention shift task, a movement detection task, and a spatial working memory task. They observed a linear decrease of MedDrive scores with driving performance of drivers over 70 in an on-road evaluation. Also, the new variant of the UFOV predicted driving performance better than the classical variant. A meta-analysis of 21 studies [87] investigated the predictive power of cognitive tests on three criteria of driving skills in older drivers: driving in real traffic, driving in a simulator, and specific driving problems. In particular, attention functions and visual tests separate good and poor older drivers in different aspects of driving behavior.

The advantage of tests that assess driving-relevant functions is their high experimental control and objectivity, as well as their easy and cost-efficient administration. There is a consensus among driving researchers that the rating of driving skills of older adults cannot rely on their performance in single sensory, motor, and cognitive tests (e.g., [88]). However, combinations of such tests have a better predictive power for driving behavior (e.g., [22,27,89]). When comparing the different studies, the tests or test combinations that are predictive of driving behavior vary considerably, which also depends on the *a priori* selection of subtests. Also, there are by now no cutoff or critical test scores to safely identify drivers with impaired driving skills and reject the unimpaired [90]. One problem that contributes to insufficient sensitivity or specificity is self-selection: mainly good drivers take part in driving studies. For example, in the study of Vaucher *et al.* [86] only 12 of the 184 participants were judged as poor drivers, and some of those scored relatively highly on the MedDrive, while some excellent drivers had a relatively low score. Any cutoff point in the summary score would have led to misallocation. This again shows clearly that even sophisticated cognitive tests, such as those combined in the Vaucher *et al.* study, are not sufficient to predict driving in real traffic, and certainly not sufficient to restrict driving.

More realistic tests use PCs or driving simulators to administer more traffic-like tasks or situations. Among these are tests such as the widely-used lane change test (LCT) [91] that focus on visuomotor abilities such as tracking and steering under dual task or distraction conditions (e.g., [43]). Other tests use even more realistic traffic scenarios to assess situation awareness (SA) and hazard perception [92,93]. Hazard perception tests can be a valid measure of crash-related driving performance [93], and appropriate measures of SA show incremental validity, even against a backdrop of a large number of cognitive variables [92]. Horswill *et al.* [94] found that hazard perception response times increased significantly with age but that this age-related increase could be explained by measures of contrast sensitivity and the useful field of view, which underlines the validity of such basic tests. As mentioned above, tests of visual motion in a driving scenario are predictive of a decrease of hazard detection in older drivers [67].

Functional deficits can be compensated for to a certain degree by experience and strategies. Many older people in general and drivers in particular have learned to use compensation strategies to keep driving skills on a sufficient level (e.g., [90,95]). However, not all drivers have learned such strategies sufficiently, and strategies may fail in complex situations. Hence the assessment of driving in real traffic in critical situations appears to be necessary.

## 4. Assessment of Driving Behavior

For the assessment of driving behavior two methods are available: the observation of real driving behavior in open-road traffic or in closed-course arrangements, or the observation of behavior in a driving simulator. Both methods have specific pros and cons.

### 4.1. Driving Behavior in Real Traffic

The observation of driving behavior in real traffic yields the largest validity [90]. Drawbacks of this approach are (i) real traffic is often not challenging enough for a sufficiently long time; and (ii) a lack of standardization and control. The first problem can be addressed by carefully choosing difficult routes and rush-hour times for driving tests, the second by using standardized driving observations. Closed-road assessments are not really sensitive in normal older drivers because they usually have no problems with handling their car and navigating real traffic.

Standardized systematic driving observations have a long tradition (*cf.* [96] for an overview). Currently different approaches are used in the United States to assess driving in seniors, e.g., the California Driving Performance Evaluation (DPE) [97], the CARA-protocol [98], the Sepulveda Road Test [99], and the Washington University Road Test (WURT) [100]. In Europe, and particularly in Germany, the TRIP protocol (Test Ride for Investigating Practical fitness-to-drive) [101] or the Wiener Fahrprobe (WF; Vienna Driving Test) [102] is frequently used.

The TRIP protocol was developed at Groningen University by the group of Brouwer [101]. It measures global driving behavior across many situations, which are distributed over a standardized route of about 35 km in length. Specifically, driving behavior is evaluated on 11 dimensions or scales with subscales. In several studies the TRIP protocol has shown high inter-rater reliability and internal consistency when used with older drivers (e.g., [103]). It is currently a standard tool for assessing driving competence.

In contrast to the TRIP protocol, the Wiener Fahrprobe [102] focuses on specific inadequate driving behavior in single traffic situations, such as too small a distance from the leading car or endangering pedestrians. Also, the Wiener Fahrprobe has sufficient inter-rater reliability and good criterion validity concerning individual accident rates (e.g., [96]).

More recently, some on-road evaluation instruments have been developed specifically for assessing older drivers. Of the four methods constructed using statistical methods, two are too complex, relying on a scoring method using over 75 items [104,105], and one was aimed at drivers with vision deficits [106]. Only the P-drive [107,108] focuses on age-related cognitive impairment and has been validated during on-road tests.

As mentioned above, it is doubtful whether critical situations for older drivers actually occur during a standardized driving assessment, even if demanding routes are chosen. Hence even poor drivers may drive inconspicuously. In addition, the performance of different drivers can hardly be compared because of unpredictable differences between the actual traffic situations and other surrounding conditions. A suitable tool for measuring driving behavior in complex and strictly controlled traffic situations is the driving simulator.

### 4.2. Behavior in the Driving Simulator

Driving simulators allow the simulation and repetition of complex traffic situations independent of daytime and weather. Moreover, they avoid risk to the drivers and other road users when encountering difficult situations in real traffic. The high control over driving situations and tasks and the exclusion of confounding variables enables a high degree of standardization between subjects and therefore allows for comparisons between subjects and groups. Driving simulators are hence a good compromise between laboratory experiments, which are far from reality, and real driving, which is hard to control [109].

Several studies confirm the validity of driving simulator data to estimate driving behavior in real traffic [110]. In particular, coping with complex situations in the simulator is related well to real driving performance in older drivers (e.g., [111]). Apart from their high cost, there are several drawbacks of simulators (e.g., [112]). First, simulators simplify the real situation and may disregard information that is present in real traffic. Moreover, the often playful character of a simulator drive may reduce the motivation of a subject to take the task seriously enough, or rather may enhance risk-taking. Finally, many older subjects get sick when driving in the simulator [113], which excludes them as subjects. A good comparison of pros and cons of driving simulator tests can be found in Caird and Horrey [114].

The different test procedures have pros and cons and should be combined to reach maximum validity concerning the driving competence of an older driver (e.g., [109]).

## 5. Measures to Improve Mobility in Older Drivers

Several measures are possible to compensate for age-related deficits and increase the driving competence of older drivers. Such measures target either the traffic or car environment or the drivers themselves.

### 5.1. Information Campaigns

Information campaigns address the broad public, giving information about factors that influence fitness to drive, or about training for driving safety. Concerning the former, the majority of older drivers already have good health-related knowledge [115]. However, there is usually a lack of knowledge about the influence of certain drugs on fitness to drive, which requires much more educational advertising.

Training for driving safety usually has specific objectives such as safe driving in winter and is administered in special driver training areas. Notwithstanding the benefit of such trainings, they only have limited additional value for older drivers. First, they focus on skills such as driver–car interactions that are unaffected in experienced older drivers; second, they often address situations that are avoided by older drivers, such as driving on snow; third, the training situations are usually much more difficult to handle in real traffic because of the presence of other road users and a more complex environment (e.g., [116]).

### 5.2. Design of Traffic and Car Environment

Certain traffic situations are particularly difficult to handle for older drivers, such as turning at complex crossroads, especially turning left across the traffic flow [117]. Those difficulties are aggravated by poor design of such traffic junctions (e.g., [116]).

Due to systematic observations of junctions, left-turning traffic is often not protected by traffic lights, or guidelines and waiting lines are missing or not recognizable [118]. Based on the abovementioned problems the elderly have with visual search and distractibility, the traffic environment at intersections should be designed to be as simple and clear as possible without distracting and traffic-irrelevant information such as advertisements. Complex areas should be clearly structured and traffic routing distinctly marked by eye-catching coloring. Also, as mentioned by road users themselves, barrier-free design and traffic infrastructure for bicyclists is important [90,118].

Also, well-designed car technology can help elderly drivers (e.g., [119]). In-vehicle information systems (IVIS), and above all route guidance systems are most helpful for the elderly in difficult situations since they reduce search. Such systems should give announcements sparsely and just in time for proper preparation. Ideally they should not give any information in critical situations to avoid overloading or distraction in older drivers [90]. However, this requires further research to develop more intelligent systems. Also important are sensors that signal distance from obstacles and parking assistants that support backing into a parking space (e.g., [116]).

### 5.3. Individual Means

Individual means address groups or individuals and focus indirectly or directly on behavioral changes of older drivers (*cf.* [116] for an overview).

#### 5.3.1. Instructions

Instruction programs provide information on possible age-related deficits, related hazards, and strategies to cope with both. However, this usually does not change accident rates, independent of drivers’ age [120]. Also, instruction programs do not change risk perception and openness to change in older drivers; older men, especially, seem to be unwilling to change their driving habits due to instruction [116]. In a study of Nasvadi and Varvrik [121], an instruction program even had a negative effect on a subset of male drivers over 75 years. As discussed by the authors, this may be due to a lack of intrinsic motivation, and a failure to implement the knowledge presented in the course. Also, those participants continued to believe that others were at fault for crashes. The authors suggest that future programs should include components that deal with recognizing culpability for driving. Finally, when compared with a simulator, training instructions do poorly [122].

#### 5.3.2. Functional Trainings

Kua *et al.* [123] reviewed the efficacy of different means in older drivers concerning driving behavior and accident rate. Instructions, physical training, and training of visual perception improved driving-relevant skills but had no effect on accident rates. However, a later review of Korner-Bitensky *et al.* [124] showed clear improvements in the driving skills of older drivers through a combination of instructions and physical retraining. Moreover, positive effects of physical training were found, while instruction alone was not effective, which confirms the abovementioned studies.

Functional training addresses either the functions underlying driving or driving behavior itself. In some studies positive effects of PC-based cognitive training on the driving competence of older drivers were reported (e.g., [125]).

Ball *et al.* [126] investigated the impact of different cognitive training (memory, reasoning, or UFOV training *vs.* no training) on the accident rate in a large sample of older drivers. The active training was composed of 10 sessions of 70 min within 5–7 weeks. Outcome was the accident involvement in a time period of six years after onset of the training. While the memory training yielded no effects, both the UFOV and the reasoning training resulted in a strongly reduced accident rate compared to the no-training group, irrespective of confounding factors such as age, gender, education, health state, and kilometers driven. In a study of Cassavaugh and Kramer [127], older subjects trained in attention, working memory, and manual control in single and dual task conditions. Apart from the usual improvements in the trained tasks, the trainees showed improvements in simulated driving. Horswill *et al.* [128] trained older drivers to recognize hazardous situations, which indeed enabled the trainees to faster anticipate such situations. In a recent overview paper, Ross *et al.* [129] stated that adequate cognitive training generally shows transfer of training to several mobility functions, including driving safety, driving difficulty, and driving cessation.

The pros of such functional trainings are the low cost, the easy administration, and the possibility of training several persons at the same time.

#### 5.3.3. Driving Training in Real Traffic

Driving in real traffic enables training for difficult situations in a real context. In the Dortmund driving training study, older drivers over 70 years showed a significant improvement of their driving competence as measured with the TRIP protocol [103]. The training consisted of 15 sessions of supervised driving on highly demanding routes. The routes contained typical problem scenarios for older drivers, such as left turns and crossroads, as well as accident blackspots. After the training the old drivers showed the driving skills of an untrained middle-aged driver. The largest improvements were seen for those drivers who showed the worst *a priori* driving performance. The improvements were still seen after 12 months. However, even when routes are carefully chosen and contain maximum challenge for older drivers (as in the Dortmund study), critical situations depend on traffic volume and time of day, so they cannot be guaranteed for each driver and each session. In addition, driving training in real traffic is costly and not risk-free.

#### 5.3.4. Driving Training in the Simulator

Driving simulators can be programmed, so critical situations can be highly controlled, repeated, and trained. An important goal for simulator training is to provide feedback and have people learn to look out for important signals. Several studies show positive effects of simulator training on driving competence in real traffic. Roenker *et al.* [130] compared simulator training and functional (UFOV) training in older drivers. The authors assessed driving performance before, directly after, and 18 months after the training both in a simulator and in real traffic. While the simulator training (but not the UFOV training) improved the behavior of older drivers at left turns and traffic lights, the UFOV training (but not the simulator training) improved divided attention. Lavallière *et al.* [131] found such real driving improvements when the simulator training was coupled with driving-specific feedback. In a recent study of Casutt *et al.* [132], older drivers were assigned either to simulator training, attention training, or a no-training control group. Before the training phase, driving performance in real traffic and cognitive performance in traffic-related tests [133] was assessed for all participants. After 10 training sessions, both tests were administered again. Both training groups improved their cognitive test performance compared to the control group, but only the group trained in the simulator showed an improvement in real driving performance. The authors assume that the advantage of the simulator training is due to its more realistic and dynamic structure, which facilitates transfer to real traffic situations, whereas functional training is more abstract and far from reality. On the other hand, as mentioned above, functional training such as UFOV appears to reduce the accident rate in older subjects. Also, compared to functional training, which only needs a PC and software, driving simulators are rare and expensive. Hence studies are needed that compare both short-term outcomes (such as divided attention skills and behavior in complex traffic situations) as well as long-term outcomes such as accident rate and driving cessation in older drivers.

## 6. Recommendations for Safe Driving in Older Age

With increasing age, certain functional deficits can impair driving skills and the interaction of the driver with the environment. Means to improve safe driving for older people address either the traffic or car environment or the individual driver. As to the environment, complex locations like crossroads should be structured as clearly as possible. This includes explicit guidance and the avoidance of any distracting stimuli such as ads at critical sites. In-vehicle information systems (IVIS) can be very helpful; in particular, navigation systems are highly important for reducing search activity and should be used routinely by older drivers when driving in unfamiliar locations. Advanced driver assistance systems (ADAS) are still in development; they may be helpful for older drivers if they reduce the mental workload at critical locations.

Concerning measures for individual drivers, instructions and training are available [93]. Instructions have little impact if not combined with training. Instruction should focus not only on the importance of vision for safe driving but also on cognitive functions. In this context the relationship between cognitive function and driving competence and the possibility of improving such functions through training as well as through learning specific strategies should be pointed out.

Based on scientific evidence, expert surveys, and discussions with older drivers, Falkenstein *et al.* [116] recommended a sequence of measures to be taken if older drivers themselves, their relatives, or the family doctor raise doubts about the driving competence of an older individual. First, important visual functions should be tested, which should include not only visual acuity but also mesopic vision (vision in relative darkness) and peripheral vision. Moreover, driving-relevant cognitive skills should be tested. Such tests should include traffic skills such as situation awareness, as well as basic cognitive functions such as divided spatial attention, visual search, and multitasking. However, poor results in tests do not predict driving performance because of compensation strategies that have been developed by many older drivers. Nevertheless, poor test results signal a certain risk. After test repetition with persistent poor results, driving observation in real traffic with complex situations is recommended. If the driving test yields clear negative results, driving training should be administered, which includes critical situations and the learning of strategies to cope with such situations. Simulator training of complex situations or PC-based training of traffic-relevant cognitive functions is promising for the improvement of driving skills or lowering of the accident rate in older drivers. However, more controlled studies are necessary to evaluate the effects of such innovative training on driving-related outcomes and their sustainability. Such a support chain could be provided by the cooperation of general practitioners (who enjoy the confidence of their patients), driving instructors, and, most importantly, traffic psychologists and occupational therapists who are trained in driver assessment and rehabilitation. General practitioners, who are usually the primary point of contact for older people outside the family, should start with visual and cognitive screening. In the case of driving-relevant diseases or cognitive impairments, they should suggest including a traffic psychologist, who then brings in a driving teacher. If driving problems are established, driving training should be suggested and administered by an occupational therapist. Such a procedure, starting with the trusting patient-physician relationship, may help older drivers to face problems and look for interventions that may help them to continue driving safely. Such cooperation is, of course, only possible when prior political and administrative policies have been established.

## 7. Summary and Conclusions

Old people need individual mobility and hence should drive their own car as long as possible. While steering is an overlearned task, interaction with traffic situations and other traffic participants is a complex task that requires a multitude of sensory, motor, and cognitive functions. Such functions tend to deteriorate in older age and may affect driving competence. Some older drivers indeed have problems with certain complex and unpredicted traffic situations. Unfortunately, the driver himself is often not the best judge of his own driving skills. To assess driving competence in old people, basic visual and cognitive functions, higher traffic-related skills, or driving behavior itself can be assessed. Functional tests should include those functions that are truly relevant for driving. Visual tests should hence include contrast sensitivity and peripheral vision. Cognitive tests should include all relevant functions such as divided attention, visual search, motion detection, and dual tasking in a comprehensive battery to improve their sensitivity for driving competence. Driving tests should preferably take place in real traffic in complex situations. Measures for improving driving competence in older people range from simple instructions to functional and simulator training as well as driving training in real traffic. Instructions alone have no reliable impact on driving competence. Driving training in real traffic with complex situations is preferred since it has been shown to improve driving skills in older people up to the level of middle-aged drivers. Functional and simulator training are promising but require more research as to their efficacy in improving driving-related outcomes in older drivers. The principal aim of all those interventions should be the promotion of mobility in older adults and hence their independent living.
